# The Effect of Okra (*Abelmoschus esculentus* (L.) Moench) Seed Extract on Human Cancer Cell Lines Delivered in Its Native Form and Loaded in Polymeric Micelles

**DOI:** 10.1155/2019/9404383

**Published:** 2019-10-21

**Authors:** Watcharaphong Chaemsawang, Weerapong Prasongchean, Konstantinos I. Papadopoulos, Garnpimol Ritthidej, Suchada Sukrong, Phanphen Wattanaarsakit

**Affiliations:** ^1^Department of Pharmaceutics and Industrial Pharmacy, Faculty of Pharmaceutical Sciences, Chulalongkorn University, 254 Phyathai Road, Pathumwan, Bangkok 10330, Thailand; ^2^Department of Biochemistry and Microbiology, Faculty of Pharmaceutical Sciences, Chulalongkorn University, 254 Phyathai Road, Pathumwan, Bangkok 10330, Thailand; ^3^THAI StemLife, 566/3 Soi Ramkhamhaeng 39 (Thepleela 1), Prachaouthit Rd, Wang Thonglang, Bangkok 10310, Thailand; ^4^Department of Pharmacognosy and Pharmaceutical Botany, Faculty of Pharmaceutical Sciences, Chulalongkorn University, 254 Phyathai Road, Pathumwan, Bangkok 10330, Thailand; ^5^Research Unit of DNA Barcoding of Thai Medicinal Plants, Faculty of Pharmaceutical Sciences, Chulalongkorn University, 254 Phyathai Road, Pathumwan, Bangkok 10330, Thailand

## Abstract

Cancer is a noncommunicable disease with a high worldwide incidence and mortality rate. The National Cancer Institute of Thailand reports increasing cumulative incidence of breast, colorectal, liver, lung, and cervical cancers, accounting for more than 60% of all cancers in the kingdom. In this current work, we attempt to elucidate the phytochemical composition of the okra (*Abelmoschus esculentus* (L.) Moench) seed extract (OSE) and study its anticancer activity, delivered in its native form as well as in the form of polymeric micelles with enhanced solubility, in three carcinoma cell lines (MCF-7, HeLa, and HepG2). The presence of flavonoid compounds in the OSE was successfully confirmed, and direct delivery had the highest cytotoxic effect on the breast cancer cell line (MCF-7), followed by the hepatocellular carcinoma (HepG2) and cervical carcinoma (HeLa) cell lines in that order, while its delivery in polymeric micelles further increased this effect only in the HepG2 cell line. The OSE's observed cytotoxic effects on cancer cell lines demonstrated a dose and time-dependent cell proliferation and migration inhibition plausibly due to VEGF production inhibition, leading to apoptosis and cell death, conceivably due to the four flavonoid compounds noted in the current study, one of which was isoquercitrin. However, in view of the latter compound's isolated effects being inferior to those observed by the OSE, we hypothesize that either isoquercitrin requires the biological synergy of any one or all of the observed flavonoids or any of the three in isolation or all in concert are responsible. Further studies are required to elucidate the nature of the three unknown compounds. Furthermore, as we encountered significant problems in dissolving the okra seed extract and creating the polymeric micelles, further studies are needed to devise a clinically beneficial delivery and targeting system.

## 1. Introduction

Cancer is a noncommunicable disease with a high worldwide incidence and mortality rate [1–3]. The National Cancer Institute of Thailand reports increasing cumulative incidence of breast, colorectal, liver, lung, and cervical cancers, accounting for more than 60% of all cancers in the kingdom. In this current work, we focus on three cancer types, breast and cervical cancer with the highest incidence in Thai women, while liver cancer has the highest cancer mortality rate in Thailand.

*Abelmoschus esculentus* (L.) Moench (okra) is a flowering plant in the Malvaceae family. Since okra pods are edible, the okra plant, a native to Africa, is planted in many tropical and subtropical regions around the world, such as Southeast Asia, the Indian subcontinent, and the Mediterranean. In folk medicine, okra is commonly used to treat gastritis. Pharmacological studies have highlighted its antioxidant, neuroprotective, antidiabetic, antihyperlipidemic, and anti-fatigue activities [4]. Okra pods have previously been shown to contain high contents of polysaccharides, polyphenols, and flavonoids. The latter two possess strong antioxidant effects [5, 6] and emanate from the okra seeds while its skin extract hardly displayed such reactions. A substance frequently mentioned in the okra seed extract is isoquercitrin, with higher bioavailability than quercetin, displaying a number of chemoprotective effects both *in vitro* and *in vivo*, against oxidative stress, cardiovascular disorders, diabetes, allergic reactions, and cancer [7]. Isoquercitrin has shown inhibition of urinary bladder and pancreatic cancer progress [8, 9], as well as colon cancer suppression [10]. Despite the above numerous mentions, there is a paucity of reports regarding the anticancer effects of okra seeds. Solubility issues pertaining to flavonoid compounds necessitate a delivery system to increase cellular uptake and cytoplasm accessibility. One of the most promising delivery systems is the polymeric micelles, due to the easiness and low cost of preparation [11–14].

The aim of the present study was to elucidate the phytochemical composition of the okra seed extract and study the anticancer activity of its flavonoid-rich fraction, delivered in its native form as well as in the form of a polymeric micelle with enhanced solubility, in three carcinoma cell lines.

## 2. Materials and Methods

### 2.1. Chemicals and Reagents

Isoquercitrin (quercetin 3-O-glucoside) of 90% purity and poloxamer 407 bioreagent grade [9003-11-6] were purchased from Sigma-Aldrich, while Dulbecco's modified Eagle's medium (DMEM) and Roswell Park Memorial Institute (RPMI) 1640 medium were purchased from Biowest. Fetal bovine serum (FBS), L-glutamax, and antibiotic/antimycotic were purchased from Invitrogen. HPLC reagents, standards, and solvents were of Analytical Reagent (AR) grade and purchased from Sigma-Aldrich. Ferric chloride reagent and Folin–Ciocalteu reagent were purchased from Merck Millipore.

### 2.2. Preparation of Okra Seed Extracts

**Figure 1:**
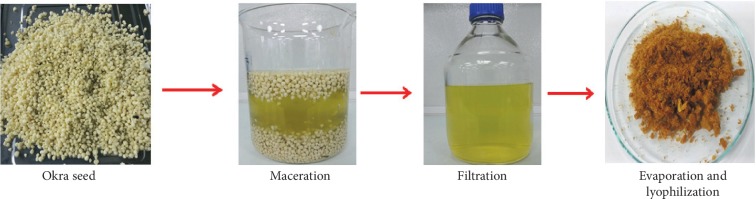
The okra seed extraction process.

Okra pods grown locally in Nakhon Pathom province in Thailand were purchased at the Pathom Mongkol market in the same province and were in a state of maturity, ready to be prepared for human consumption. The method of extraction used in our work was modified from the method of Adetuyi and Ibrahim [15]. The seeds were separated from their fruit before maceration with 95% ethanol for 24 hrs. The macerated solution was filtered and evaporated using an evaporator. The concentrate was dried using a lyophilizer (Labconco) (Figure 1). The crude okra seed extract was stored at −20°C without exposure to light until used.

### 2.3. Qualitative and Quantitative Analysis of Phytochemicals

The composition of the extract was screened by using a partition method before testing as per the below described method. The okra seed extract (OSE) was partitioned with hexane : ethanol : water in the ratio 50 : 45 : 5. The ethanol layer was further partitioned with dichloromethane : ethanol : water in the ratio 50 : 30 : 20. Each fraction was let to evaporate, (OSE-Hex, OSE-DCM, and OSE-EtOH) before chemical testing.

#### 2.3.1. Phytochemical Screening

Phytochemical screening of okra seed extract, ethanol, and dichloromethane and petroleum fraction of okra seeds were performed for the qualitative detection of different phytochemicals such as alkaloids, flavonoids, saponins, glycosides, phenols, and tannin. All phytochemical tests were performed according to the standed methods described by Samejo et al. [16] and Maria et al. [17].

#### 2.3.2. Determination of Total Phenolic Content

Total phenolic contents (TP) in different seed extracts of okra seeds were determined using a modified Folin–Ciocalteu (FC) colorimetric method [18–20]. In short, 25 *μ*l of the extract was mixed with 75 *μ*l deionized water in 96-well plates, and then, 1 : 1 FC reagent was added and incubated in the dark for 6 minutes. Following this, 100 *μ*l of 7.5% Na_2_CO_3_ was added, and the solution was incubated in the dark for another 90 min. The phenolic content is then measured using a microplate reader at 765 nm wavelength carried out using a standard solution of gallic acid, reported as gallic acid equivalents (GAE)/gram dry weight extract.

#### 2.3.3. Determination of Total Flavonoid Content

The total flavonoid content (TF) was determined spectrophotometrically [18, 21]. A sample extract of 20 *μ*l was mixed with 60 *μ*l ethanol in 96-well plates. Then, 4 *μ*l of 10% AlCl_3_ and 4 *μ*l of 1 M potassium acetate were added with 112 *μ*l deionized water. The solution was incubated in the dark for 45 minutes. The total flavonoid content was measured afterwards using a microplate reader at 415 nm wavelength. A calibration curve is prepared using a standard solution of quercetin, reported as quercetin equivalents (QE)/gram dry weight extract.

#### 2.3.4. Determination of Total Polysaccharide Content

The total polysaccharide (TPS) content was determined with phenol-sulfuric acid method modified from the method of Masuko et al. [22]. In brief, 600 *μ*l of extract was mixed with 100 *μ*l of 5% phenol solution. Then, 3 ml of concentrated sulfuric acid was added and left for 10 minutes. Following this step, the solution was shaken well and left for 30 minutes. The TPS content was measured using UV-spectrophotometry at 490 nm wavelength compared with a standard curve obtained from the glucose standard.

#### 2.3.5. Determination of Isoquercitrin

Isoquercitrin quantitative analysis was conducted according to a modified method as described by Seal [23] on a Shimadzu HPLC/DAD using a 250 mm (Kinetex) column and gradient injection of the mobile phase of 1% of acetic acid (solvent A): acetonitrile (solvent B) at a flow rate of 0.7 ml/min and detected at a wavelength 353 nm ( Table 1).

### 2.4. Cell Cultures

#### 2.4.1. Cell Lines and Cultures

The human breast cancer cell line (MCF-7), human hepatocellular carcinoma (HepG2), and human cervical carcinoma cell line (HeLa) were purchased by our institution from ATCC. The seeding medium for each cell type was as follows: MCF-7 culture in DMEM with 10% fetal bovine serum containing 1% antibiotic/antimycotic and 1% L-glutamax; HepG2 culture in RPMI-1640 medium with 10% fetal bovine serum containing 1% antibiotic/antimycotic and 1% L-glutamax; and HeLa culture in DMEM with 10% fetal bovine serum containing 1% antibiotic/antimycotic. Cells were cultured at 37°C in 5% CO_2_. Media culture was changed every 3 days.

#### 2.4.2. Cytotoxic Assay

The extract was dissolved in complete medium containing 1% DMSO to clear solution. The cytotoxicity test of cancer cells uses a PrestoBlue assay test method by seeding cells in 96-well plates and incubating for 24 hours. The tests are performed at 24 and 48 hours and measured with a microplate reader at excitation/emission 560/590 nm. Each OSE fraction was compared to the crude okra seed extract. The most effective OSE part in the cytotoxicity assay was then further compared to standard isoquercitrin equivalent to isoquercitrin found in the 1,000 *μ*g current okra seed extract.

#### 2.4.3. Cell Scratch Assay

Cells plated onto 12-well culture plates in complete medium monolayers, were manually scratched with a yellow pipette tip, followed by washing with PBS. The monolayers were then incubated with complete medium of different concentrations of the okra seed extract at 37°C for 24, 48, and 72 hours. The monolayers were photographed at 0 hours and 24 hours after scratching using an inverted microscope (Olympus, Tokyo, Japan) to capture the size of the scratch. The experiments were performed in triplicate for each treatment group.

#### 2.4.4. Cell Invasion Assay

The Boyden chamber migration test was used to study cell invasion. The 8.0 *μ*m PET filters 24-well cell culture inserts (Falcon, USA) were coated with Matrigel (Corning) and incubated for 1 h at 25°C to form a thin layer on the filter surface. The complete medium was added to the lower compartment of the chamber. After that, the wells on the upper chamber were seeded with 2 × 10^4^ cell/well, and okra seed extract was added at different concentrations ranging from 100 to 500 *μ*g/ml in low (1%) FBS medium. After 48 hours, noninvasive cells were wiped in the upper chamber using a cotton bud; lower and upper chamber were washed three times with PBS (pH 7.4) and then fixed with 3% glutaraldehyde in PBS and stained with 0.5% crystal violet in PBS. Later, the cells were lysed by using 10% acetic acid, and the quantity of cells was analyzed by using a microplate reader at a wavelength of 590 nm.

#### 2.4.5. Cell Apoptosis

The cells were cultured in a 6-well cell culture plate at a seeding density of 1 × 10^6^ cells/well incubated for 24 h at 37°C in 5% CO_2_ before treating with the extract. The cells were cultured in a 6-well cell culture plate at a seeding density of 1 × 10^6^ cells/well. After the cells were treated with different doses of the okra seed extract for 48 h, the cells were trypsinized and a single cell suspension was prepared. Then, the cells were stained using Annexin V and 7ADD (Guava Nexin Reagent, Merck Millipore), which were then analyzed by flow cytometry (Guava, Merck Millipore).

#### 2.4.6. Antiangiogenesis

Cancer cells were seeded on 12-well plates and incubated for 24 h at 37°C in 5% CO_2_. After that, the old medium was washed, and okra seed extract was added at different concentrations ranging from 25 to 1,000 *μ*g/ml in medium and incubated for 24 and 48 hours. After that, the supernatant was collected and centrifuged to analyze the quantity of vascular endothelial growth factor (VEGF) by using a sandwich ELISA (VEGF ELISA kit, Abcam). The obtained supernatant was placed in a 96-well plate kit according to the company protocol and analyzed for VEGF by using the microplate reader at a wavelength of 450 nm.

### 2.5. Preparation and Characterization of Okra Seed Extract Loaded in Polymeric Micelles

#### 2.5.1. Preparation of OSE-Loaded Micelles

OSE-loaded micelles were prepared by the thin-film hydration method. In brief, 50 mg of okra seed extract and poloxamer 407 were dissolved in ethanol in a round bottom flask. Each formulation used different concentrations of poloxamer 407, ranging between 50 and 1,000 mg of poloxamer 407. Then, the solvent was made to evaporate to a solid film in a solvent evaporator at 50°C. After that, the dried film was dehydrated with 10 ml deionized water and was sonicated for 15 minutes. The solution was filtrated by using a 0.45 *μ*m syringe filter.

#### 2.5.2. Characterization of OSE-Loaded Micelles

Particle size and entrapment efficiency were studied. Diameter, polydispersity index (PDI), and zeta potential of micelles were determined by dynamic light scattering using a Malvern Nano ZS (Malvern Instruments Ltd., Worcestershire, UK). Entrapment efficacy was evaluated by disrupting the micelles in acetonitrile (Merck Millipore). The okra seed extract was quantified by UV-visible spectrophotometry at 353 nm.

#### 2.5.3. Anticancer Activity of OSE-Loaded Micelles

Optimum dose (for micellar formation) OSE-loaded micelles were tested for cancer cell cytotoxicity using the PrestoBlue assay method as described in Section 2.4.2. OSE-loaded micelles and blank micelles were compared to each other after dilution with cell culture medium to a concentration comparable with okra seed extracts of 500 *μ*g/ml.

### 2.6. Statistical Analysis

Data were expressed as mean ± standard deviation (SD). The statistical differences between the means were determined using one-way ANOVA. The differences were considered significant when the probability value obtained was found to be less than 0.05 (*p* < 0.05).

## 3. Results and Discussion

From the results of the phytochemical tests, it was deduced that the okra seed extract contained flavonoid glycosides, positive for the Molisch test, as well as for the FeCl_3_, Shinoda, and sodium hydroxide tests (Table 2).

The quantitative analysis results were consistent with the results of phytochemical screening, meaning that the substances found in the okra seed extract were as in the group of flavonoid glycosides. The total phenolic (TP) and total flavonoid (TF) contents were found highest in okra seed extract (Table 3).

**Figure 2:**
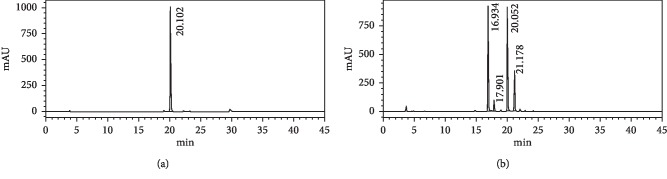
Chromatogram from HPLC for (a) standard isoquercitrin and (b) okra seed extract.

We observed that the major constituents were detected in the ethanol layer, in concentrations close to the okra seed extract albeit lower. In addition, the TPS content, an important part of flavonoid glycoside constituent, was relatively high at 36.73% in the okra seed extract. The HPLC chromatogram showed four semipolar peaks consistent with isoquercitrin and three other phenolic compounds appearing in the early stages that were not present when partitioned using hexane and dichloromethane (Figure 2).

### 3.1. Cytotoxicity

**Figure 3:**
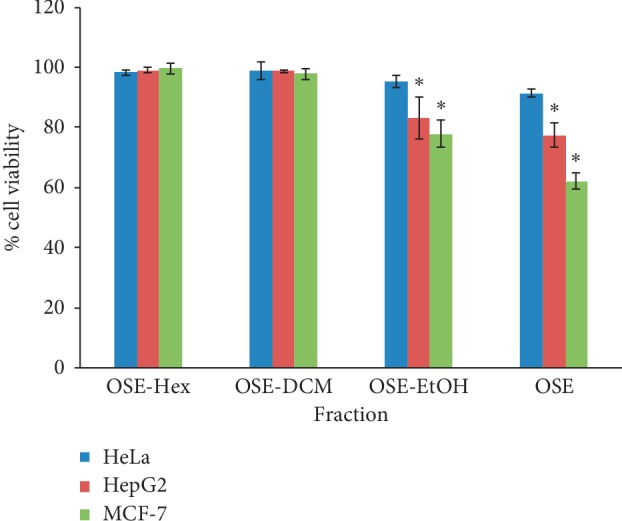
Cell viability at 48 hours: comparison between the okra seed extract (OSE) and fractions. Hexane (OSE-Hex), dichloromethane (OSE-DCM), and ethanol (OSE-EtOH). Mean ± SD (*n* = 3). (^*∗*^*p* < 0.05 versus nontreated controls at 48 hrs).

When the cytotoxic effect of the OSE and its various fractions was tested on the three cancer cell lines, we observed no effects of the hexane (OSE-Hex) and dichloromethane (OSE-DCM) fractions compared to the controls. When the ethanol fraction (OSE-EtOH) and the okra seed extract were applied, the latter had a more significant cytotoxic effect, possibly due to the synergy of various substances in preventing cancer growth (Figure 3) [24–26]. In the present study, the onset of the okra seed extract's cytotoxic effect appeared at 48 hours without any visible effects at 24 hours (data not shown), indicating a possibly chronically toxic effect.

**Figure 4:**
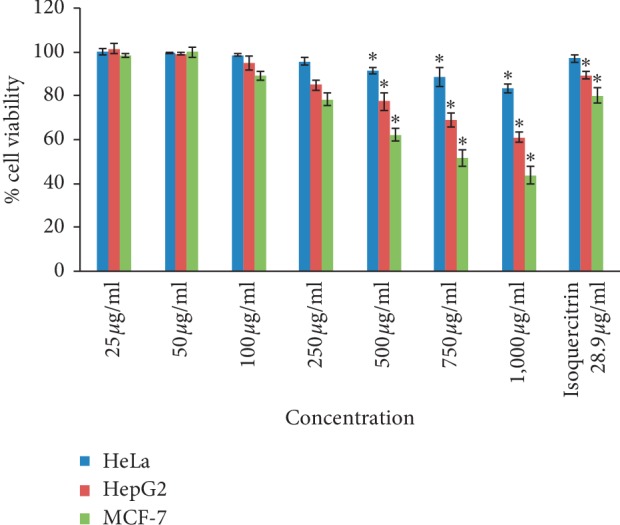
Dose-dependent effect of the okra seed extract (OSE) on cell viability at 48 hrs. Mean ± SD (*n* = 3). ^*∗*^*p* < 0.05 versus nontreated controls.

A dose-dependent effect of the okra seed extract was noted, commencing already at the 100 *μ*g/ml dose. The cytotoxic effects were highest on the growth of the MCF-7 cell lines, followed by the HepG2 (Figure 4). The effect was minimal for the HeLa cell line indicating resistance to the extract as reported by Jarial et al. [27] for both quercetin and rutin [24, 25]. Interestingly, the effects of standard isoquercitrin were inferior to the okra seed extract. In the MCF-7 cell line, an OSE concentration of 250 *μ*g/ml, containing not more than 7.2 *μ*g/ml of isoquercitrin, was as cytotoxic as the equivalent of 28.9 *μ*g/ml pure isoquercitrin. When an OSE concentration of 1000 *μ*g/ml containing the equivalent of 28.9 *μ*g/ml pure isoquercitrin was used, we observed a potentiated cytotoxic effect for the OSE resulting in 43.6% MCF-7 cell viability compared to 79.9% for pure isoquercitrin (Table 4). We hypothesize that the remaining three unknown flavonoids observed in the HPLC chromatogram (Figure 2) separately or in synergy with isoquercitrin are responsible for the OSE's observed potentiated cytotoxic effects.

### 3.2. Cell Migration and Cell Invasion

**Figure 5:**
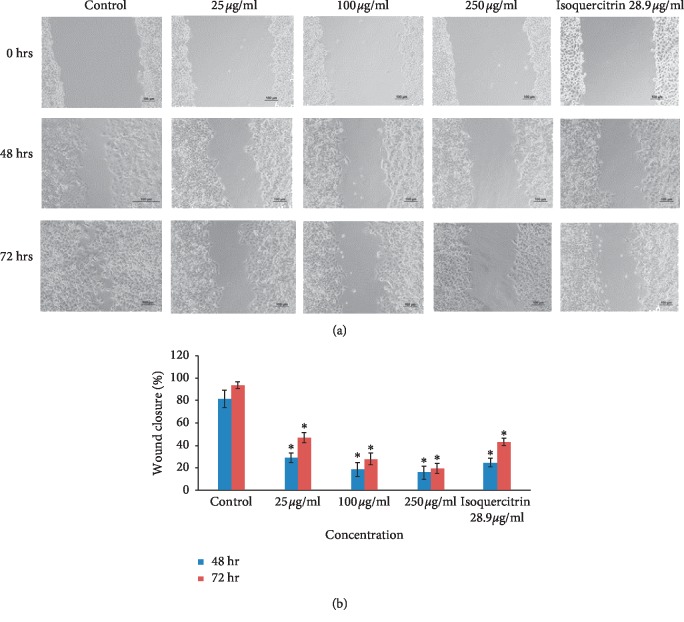
(a) Cell migration (HeLa). (b) Cell migration (HeLa). Results are shown as mean ± SD (*n* = 3). ^*∗*^*p* < 0.05 versus nontreated controls at the same time point.

**Figure 6:**
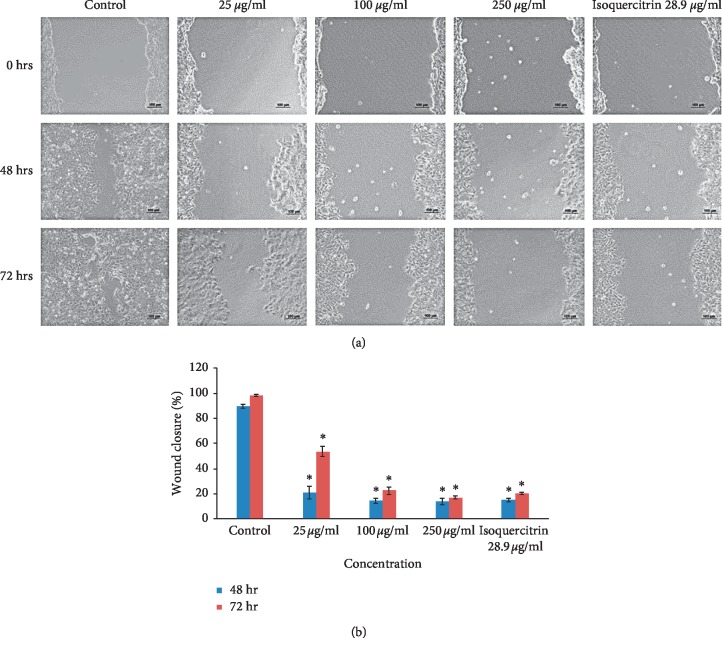
(a) Cell migration (MCF-7). (b) Cell migration (MCF-7). Results are shown as mean ± SD (*n* = 3). ^*∗*^*p* < 0.05 versus nontreated controls at the same time point.

**Figure 7:**
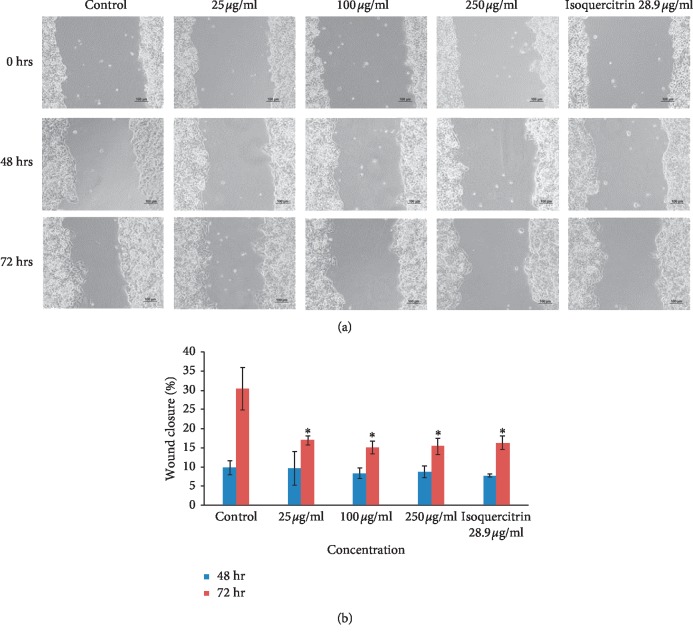
(a) Cell migration (HepG2). (b) Cell migration (HepG2). Results are shown as mean ± SD (*n* = 3). ^*∗*^*p* < 0.05 versus nontreated controls at the same time point.

The okra seed extract displayed inhibition of migration in two of the three cancer cell lines (MCF-7 and HeLa) in the cell scratch assay. The OSE extract's effects on migration inhibition in the HeLa cell line (Figures 5(a) and 5(b); Table 5) was already evident at 25 *μ*g/ml concentrations with a wound closure of 28.67 ± 4.25% compared to controls which showed a wound closure of up to 81.50 ± 7.87%. Similar results were noted in the MCF-7 cell line beginning at OSE concentrations of 100 *μ*g/ml (Figures 6(a) and 6(b); Table 5). Pure isoquercitrin showed similar albeit weaker effects compared to the crude extract. The HepG2 cells grow and divide in groups in overlapping layers; hence, the split and side of the heel appears less noticeable (Figures 7(a) and 7(b); Table 5) when the HepG2 cells were scratched while the MCF-7 prefers to divide horizontally into a monolayer rather than overlapping.

**Figure 8:**
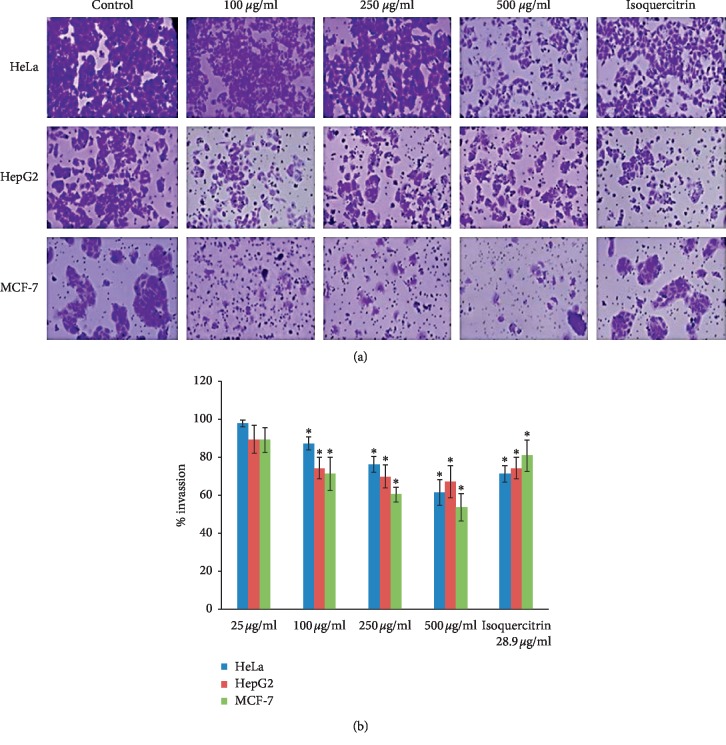
(a) Cell invasion inhibition. (b) Cell invasion inhibition. Results are shown as mean ± SD (*n* = 3). ^*∗*^*p* < 0.05 versus nontreated controls.

The Boyden chamber migration test revealed dose-dependent effects on all 3 cell lines and all 3 doses of the okra seed extract, already appearing at the lowest dose tested (Figures 8(a) and 8(b); Table 6). At all doses, the MCF-7 cell line exhibited the highest level of invasion inhibition followed by the HepG2 cells and HeLa cells, respectively. Isoquercitrin alone used as control resulted in similar effects, in the inverse order of effect on the three cell lines. Our results are in agreement with the findings of Dai et al. [28] and Zhang et al. [29].

### 3.3. Cell Apoptosis

Earlier in the results section of current study, we evaluated the okra seed extract's cytotoxic effect and reported that such effects were dose and time-dependent and highest for the MCF-7 cell lines, followed by the HepG2 while the effect was minimal for the HeLa cell line indicating resistance to the extract. The incubation with the extract showed a significant effect on the apoptosis assay.  Table 7 shows that cells treated with 25, 50, 100, 250, 500,750, and 1,000 *μ*g/mL okra seed extract for 48 h resulted in a significant increase in the percentage of apoptotic cells (early and late apoptosis) in a manner consistent with its cytotoxic effects. Thus, the MCF-7 cell line was the most responsive to induction of early and late apoptosis in a dose-dependent manner already at low doses, but slightly declined at the highest dose. Similar albeit weaker effects were noted for the HeLa and HepG2 cell lines. The results are consistent with numerous studies that found that substances in the flavonoid group may induce apoptosis of cancer cells [30–32]. In addition, different types of flavonoids may exhibit differential effects in a multitude of cancer cells, such as flavonoids extracted from propolis may induce more apoptosis in breast cancer than in colon cancer [33]. The chemical structure of different flavonoids may affect their antiradical activity, and thus induce differential effects on cancer cell inhibition [34].

### 3.4. Antiangiogenesis

In the present study, we observed that the OSE exerted its VEGF inhibition in a dose-depended manner on all 3 cell lines both at 24 and 48 hours, but this effect was only significant at 48 hours in accordance with previous reports on flavonoids and isoquercitrin (Table 8) [35, 36].

### 3.5. Polymeric Micelles

**Figure 9:**
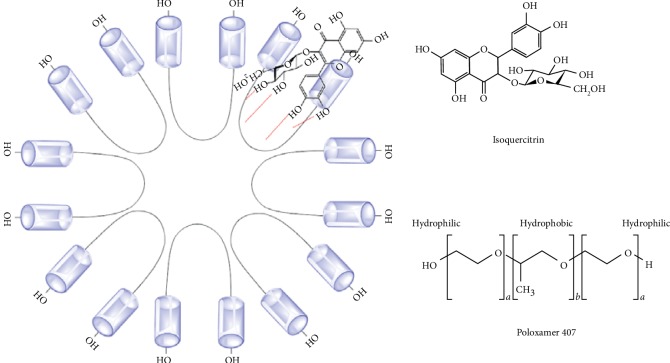
Possible location of isoquercitrin in micelles.

Poloxamer 407 is a nonionic triblock copolymer. The structure consists of 3 parts; the flanks are made by two hydrophilic chains of polyoxyethylene and a middle hydrophobic chain of polyoxypropylene. The structure is bent into the U-shape when poloxamer 407 forms the micelles, with the hydrophobic part facing inwards in the micelle and the hydrophilic part facing outwards (Figure 9). Our working hypothesis on the micelle interface is that the hydroxyl group of the flavonoid glycoside loaded from our extract will form a hydrogen bond with the poloxamer's oxygen atom causing it to be entrapped into the micelle as presented by Chat et al. [37].

The okra seed extract was loaded to the polymeric micelles using poloxamer 407 as the micellar. The optimum dose for poloxamer 407 was shown to be 50 : 500 as other ratios resulted in either small or inefficient micelles (Table 9). When the poloxamer 407 concentration increased, the polydispersity index (PDI) increased too, causing densely packed micelle formation in various forms and shapes and inadequate entrapment efficacy.

**Figure 10:**
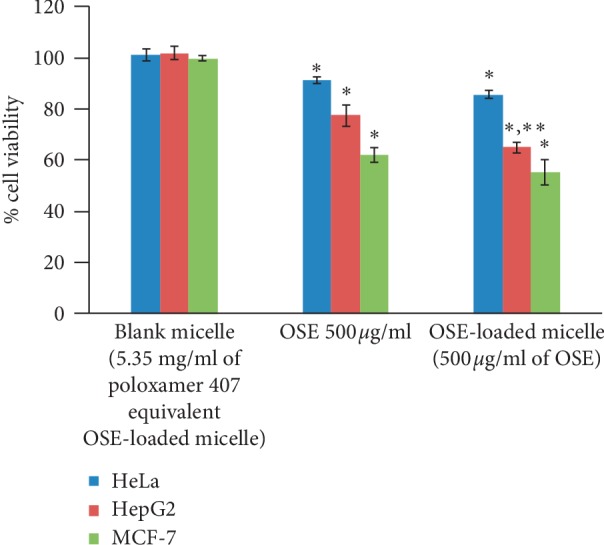
Cytotoxic activity of okra seed extract-loaded polymeric micelles. Results are shown as mean ± SD (*n* = 3). ^*∗*^*p* < 0.05 versus nontreated controls and ^*∗*^*p* < 0.05 versus OSE group.

The blank polymeric micelles did not exert any cytotoxic effect to any of the cancer cell lines. When okra seed extract was added to the polymeric micelles, water solubility increased and cytotoxicity was enhanced (Figure 10, Table 10). The increased water solubility might increase the extract's penetration rate into the cells, thereby inhibiting their growth. However, the polymeric micelle delivery system's cytotoxicity increase was only significant in the HepG2 cell line with little change in the MCF-7 and HeLa lines (Figure 10, Table 10). Previous studies reported that efflux pumps and *p*-glycoproteins are localized on the cell membrane of breast cancer cells and help transport substances in and out of the cells, potentially rending them resistant to chemotherapy [38]. Similar reports have been published on cervical cancer [39, 40]. The polymeric micelle delivery system used in the present study was an initial prototype aimed to increase the solubility of the okra seed extract. Future studies are in order to devise more specific drug-targeting system to cancer cells, such as conjugation to folic acid [41, 42].

## 4. Conclusion

In the present study, we successfully confirmed the presence of flavonoid compounds in the okra plant seed extract and evaluated the extract's effect on the three cancer cell lines delivered in its native form as well as in the form of polymeric micelles. The direct delivery of okra seed extract had the highest cytotoxic effect on the breast cancer cell line (MCF-7), followed by the hepatocellular carcinoma (HepG2) and cervical carcinoma (HeLa) cell lines in that order, while its delivery in a polymeric micelle further increased this effect only in the HepG2 cell line. The okra seed extract's effect demonstrated a dose and time-dependent cell proliferation and migration inhibition plausibly due to VEGF production inhibition, leading to apoptosis and cell death. The observed OSE effects on cancer cell lines may stem from the four flavonoid compounds in the current study, one of which was isoquercitrin. However, in view of the latter compound's isolated effects being inferior to those observed by the OSE, we hypothesize that either isoquercitrin requires the biological synergy of any one or all of the observed flavonoids or any of the three in isolation or all in concert are responsible. Further studies are required to elucidate the nature of the three unknown compounds. Furthermore, as we encountered significant problems in dissolving the okra seed extract and creating the polymeric micelles, further studies are needed to devise a clinically beneficial delivery and targeting system.

## Figures and Tables

**Table 1:** Gradient condition for HPLC analysis of the okra seed extract.

Time (min)	Pump A: 1% acetic acid	Pump B: acetonitrile
0	90	10
28	60	40
39	40	60
50	10	90
55	90	10

**Table 2:** Phytochemical screening.

	OSE-Hex fraction	OSE-DCM fraction	OSE-EtOH fraction	Okra seed extract
FeCl_3_ test, phenolics	−	−	+	+
Shinoda test, flavonoids	−	−	+	+
Sodium hydroxide test, flavonoids	−	−	+	+
Molisch test, glycosides	−	−	+	+
Dragendorff's test, alkaloids	−	−	−	−
Foam test, saponins	−	−	−	−
Gelatin test, tannins	−	−	−	−

**Table 3:** Analysis of substance content in the okra seed extract according to partition fractions.

	OSE-Hex fraction	OSE-DCM fraction	OSE-EtOH fraction	Okra seed extract
Total phenolic (TP) content	—	—	46.51 ± 5.61 mg GAE/g extract	56.66 ± 1.88 mg GAE/g extract
Total flavonoid (TF) content	—	—	41.71 ± 10.88 mg QE/g extract	44.07 ± 7.03 mg QE/g extract
Total polysaccharide (TPS) content	—	—	31.74 ± 1.23%	36.73 ± 2.41%
Isoquercitrin content	—	—	2.68 ± 1.92%	2.89 ± 1.64%

**Table 4:** Cell viability percentages (%) compared to controls at 48 hrs.

	HeLa (%)	HepG2 (%)	MCF-7 (%)
25 *μ*g/ml	100.04 ± 1.23	101.40 ± 2.27	98.60 ± 0.84
50 *μ*g/ml	99.38 ± 0.30	99.19 ± 0.38	99.90 ± 2.47
100 *μ*g/ml	98.64 ± 0.63	94.85 ± 3.29	89.07 ± 2.09^*∗*^
250 *μ*g/ml	95.75 ± 1.98	84.78 ± 2.24^*∗*^	78.35 ± 3.09^*∗*^
500 *μ*g/ml	91.40 ± 1.30^*∗*^	77.41 ± 4.11^*∗*^	62.11 ± 2.82^*∗*^
750 *μ*g/ml	88.56 ± 4.44^*∗*^	68.72 ± 3.21^*∗*^	51.39 ± 3.78^*∗*^
1,000 *μ*g/ml	83.38 ± 1.81^*∗*^	60.97 ± 2.26^*∗*^	43.60 ± 4.13^*∗*^
Isoquercitrin 28.9 *μ*g/ml	97.10 ± 1.70	89.38 ± 1.84^*∗*^	79.99 ± 3.43^*∗*^

Results are shown as mean ± SD (*n* = 3). ^*∗*^*p* < 0.05 versus nontreated controls.

**Table 5:** Percentage wound closure in the cell migration test compared on day 0.

		Control	25 *μ*g/ml	100 *μ*g/ml	250 *μ*g/ml	Isoquercitrin 28.9 *μ*g/ml
48 hr	HeLa	81.50 ± 7.87	28.67 ± 4.25^*∗*^	18.44 ± 6.14^*∗*^	15.77 ± 5.98^*∗*^	24.64 ± 3.92^*∗*^
	HepG2	9.77 ± 1.83	9.71 ± 4.33	8.35 ± 1.33	8.71 ± 1.56	7.75 ± 0.38
	MCF-7	89.70 ± 1.43	20.80 ± 4.86^*∗*^	14.51 ± 2.11^*∗*^	13.98 ± 2.48^*∗*^	14.99 ± 1.69^*∗*^

72 hr	HeLa	93.73 ± 2.97^*∗*^	46.92 ± 4.60^*∗*^	27.73 ± 5.25^*∗*^	19.48 ± 4.69^*∗*^	42.95 ± 3.36^*∗*^
	HepG2	30.45 ± 5.60^*∗*^	16.97 ± 1.14^*∗*^	15.05 ± 1.67^*∗*^	15.51 ± 2.18^*∗*^	16.30 ± 1.75^*∗*^
	MCF-7	98.14 ± 0.95^*∗*^	53.69 ± 4.25^*∗*^	22.38 ± 2.91^*∗*^	16.74 ± 1.18^*∗*^	20.25 ± 0.93^*∗*^

Results are shown as mean ± SD (*n* = 3). ^*∗*^*p* < 0.05 versus nontreated controls.

**Table 6:** Percentage cell invasion compared with controls.

	HeLa	HepG2	MCF-7
25 *μ*g/ml	98.01 ± 1.82	89.67 ± 7.30	89.25 ± 6.75
100 *μ*g/ml	87.34 ± 3.56^*∗*^	74.32 ± 5.64^*∗*^	71.46 ± 8.79^*∗*^
250 *μ*g/ml	76.25 ± 4.12^*∗*^	70.12 ± 6.15^*∗*^	60.63 ± 3.83^*∗*^
500 *μ*g/ml	61.73 ± 6.87^*∗*^	67.35 ± 8.47^*∗*^	53.71 ± 7.14^*∗*^
Isoquercitrin 28.9 *μ*g/ml	71.43 ± 4.24^*∗*^	74.41 ± 5.72^*∗*^	81.06 ± 8.27^*∗*^

Results are shown as mean ± SD (*n* = 3). ^*∗*^*p* < 0.05 versus nontreated controls.

**Table 7:** Percentages of apoptotic cells for different doses of the okra seed extract in each cell line.

		Control	25 *μ*g/ml	50 *μ*g/ml	100 *μ*g/ml	250 *μ*g/ml	500 *μ*g/ml	750 *μ*g/ml	1,000 *μ*g/ml	Isoquercitrin 28.9 *μ*g/ml
HeLa	Early apoptotic	1.13 ± 0.28	4.13 ± 0.13^*∗*^	3.92 ± 0.72^*∗*^	4.20 ± 0.18^*∗*^	6.18 ± 0.38^*∗*^	6.41 ± 0.23^*∗*^	6.69 ± 0.63^*∗*^	6.55 ± 0.13^*∗*^	5.51 ± 0.22^*∗*^
	Necrosis	0.40 ± 0.11	3.57 ± 0.43^*∗*^	5.23 ± 1.03^*∗*^	4.26 ± 0.23^*∗*^	5.18 ± 0.91^*∗*^	5.89 ± 0.17^*∗*^	7.85 ± 0.62^*∗*^	8.38 ± 0.06^*∗*^	3.87 ± 0.08^*∗*^

HepG2	Early apoptotic	0.38 ± 0.02	3.21 ± 0.09^*∗*^	3.97 ± 0.81^*∗*^	5.19 ± 0.21^*∗*^	5.23 ± 0.45^*∗*^	5.36 ± 0.24^*∗*^	12.59 ± 1.12^*∗*^	10.59 ± 0.21^*∗*^	5.83 ± 0.50^*∗*^
	Necrosis	0.32 ± 0.03	1.73 ± 0.11^*∗*^	1.67 ± 0.19^*∗*^	1.72 ± 0.08^*∗*^	2.00 ± 0.12^*∗*^	2.19 ± 0.20^*∗*^	3.47 ± 0.38^*∗*^	8.66 ± 0.58^*∗*^	3.22 ± 0.32^*∗*^

MCF-7	Early apoptotic	1.68 ± 0.25	5.37 ± 0.83^*∗*^	5.60 ± 2.46^*∗*^	12.63 ± 0.12^*∗*^	15.21 ± 1.14^*∗*^	15.62 ± 2.47^*∗*^	21.78 ± 6.08^*∗*^	19.34 ± 2.33^*∗*^	3.11 ± 0.73^*∗*^
	Necrosis	1.21 ± 0.05	3.34 ± 0.58	3.29 ± 0.61	4.60 ± 0.73^*∗*^	3.87 ± 1.72^*∗*^	5.52 ± 0.47^*∗*^	7.84 ± 1.77^*∗*^	8.05 ± 2.16^*∗*^	3.94 ± 0.40

Results are shown as mean ± SD (*n* = 3). ^*∗*^*p* ≤ 0.05 compared with controls.

**Table 8:** VEGF content analysis (pg/ml).

		Control	100 *μ*g/ml	250 *μ*g/ml	500 *μ*g/ml	Isoquercitrin 28.9 *μ*g/ml
HeLa	24 hours	125.96 ± 7.14	128.19 ± 51.24	113.00 ± 17.46	92.26 ± 14.50	121.76 ± 23.91
	48 hours	240.41 ± 14.67	184.48 ± 7.14^*∗*^	141.15 ± 2.79^*∗*^	141.89 ± 4.84 ^*∗*^	168.26 ± 14.18^*∗*^

HepG2	24 hours	253.74 ± 6.12	233.74 ± 5.48	178.56 ± 10.72^*∗*^	158.56 ± 27.31^*∗*^	193.61 ± 19.04^*∗*^
	48 hours	543.37 ± 23.74	278.93 ± 3.91^*∗*^	267.81 ± 9.45^*∗*^	190.41 ± 8.98 ^*∗*^	263.24 ± 8.16^*∗*^

MCF-7	24 hours	205.96 ± 21.92	193.74 ± 57.42	157.44 ± 46.20	129.30 ± 9.86	164.92 ± 21.83
	48hours	302.26 ± 12.64	225.89 ± 15.75^*∗*^	145.22 ± 11.76^*∗*^	137.81 ± 11.56^*∗*^	178.48 ± 15.62^*∗*^

Results are shown as mean ± SD (*n* = 3). ^*∗*^*p* ≤ 0.05 compared to controls.

**Table 9:** Physical properties of okra seed extract polymeric micelle formation.

Ratio (extract: poloxamer)	Size (nm)	PDI	Zeta potential	% EE
50 : 50	391.63 ± 43.52	0.36 ± 0.04	−9.54 ± 3.42	79.82 ± 0.29
50 : 100	404.03 ± 36.46	0.42 ± 0.08	−15.00 ± 5.10	83.57 ± 0.75
50 : 200	442.72 ± 72.43	0.44 ± 0.05	−15.27 ± 3.88	86.56 ± 0.54
50 : 500	190.23 ± 46.96	0.34 ± 0.05	−14.70 ± 4.88	93.43 ± 2.45
50 : 1,000	167.57 ± 110.58	0.99 ± 0.01	−16.02 ± 6.72	86.88 ± 1.93

**Table 10:** Cytotoxic activity of okra seed extract-loaded polymeric micelles.

	Blank micelle	OSE 500 *μ*g/ml	OSE-loaded micelle (500 *μ*g/ml of OSE)
HeLa	101.27 ± 2.42	91.40 ± 1.30^*∗*^	85.57 ± 1.55^*∗*^
HepG2	101.80 ± 2.52	77.41 ± 4.11^*∗*^	65.01 ± 2.18^*∗*^, ^*∗∗*^
MCF-7	99.72 ± 1.03	62.15 ± 2.82^*∗*^	55.24 ± 4.88^*∗*^

Results are shown as mean ± SD (*n* = 3). ^*∗*^*p* < 0.05 versus nontreated controls.

## Data Availability

The raw data used to support the findings of this study are currently under embargo while the research findings are commercialized. Requests for data, 12 months after publication of this article, will be considered by the corresponding author.
